# MRI Reporter Genes for Noninvasive Molecular Imaging

**DOI:** 10.3390/molecules21050580

**Published:** 2016-05-18

**Authors:** Caixia Yang, Rui Tian, Ting Liu, Gang Liu

**Affiliations:** State Key Laboratory of Molecular Vaccinology and Molecular Diagnostics & Center for Molecular Imaging and Translational Medicine, School of Public Health, Xiamen University, Xiamen 361102, China; caixiacx@outlook.com (C.Y.); tr_xmu@163.com (R.T.); tingliu20072008@yahoo.com (T.L.)

**Keywords:** reporter gene, MRI, molecular imaging, multimodal imaging

## Abstract

Magnetic resonance imaging (MRI) is one of the most important imaging technologies used in clinical diagnosis. Reporter genes for MRI can be applied to accurately track the delivery of cell in cell therapy, evaluate the therapy effect of gene delivery, and monitor tissue/cell-specific microenvironments. Commonly used reporter genes for MRI usually include genes encoding the enzyme (e.g., tyrosinase and β-galactosidase), the receptor on the cells (e.g., transferrin receptor), and endogenous reporter genes (e.g., ferritin reporter gene). However, low sensitivity limits the application of MRI and reporter gene-based multimodal imaging strategies are common including optical imaging and radionuclide imaging. These can significantly improve diagnostic efficiency and accelerate the development of new therapies.

## 1. Introduction

Molecular imaging includes several imaging modalities such as positron emission tomography (PET), single photon emission computed tomography (SPECT), computed tomography (CT), optical imaging (OI), ultrasound, and magnetic resonance imaging (MRI) [1,2]. MRI offers high spatial resolution and it can simultaneously obtain anatomic and physiological information [3]. The use of MRI probes has significantly improved the signal-to-noise ratio of MRI, and novel reporter gene imaging would further increase its sensitivity and specificity. MRI reporter gene imaging can longitudinally monitor the processes (e.g., cell delivery, gene expression, *et al.*) in living organisms by visualizing the levels of exogenous or endogenous gene expression, specific signal transduction pathways, nuclear receptor activities, or protein–protein interactions [4,5]. Using this noninvasive imaging technology, the total number of animals is greatly reduced, and the results are more reliable because each animal is its own control [6].

Reporter gene imaging is an indirect method to detect gene expression [5,7]. The expression products include transporters, receptors, enzymes, metalloproteins, *etc.*, which can specifically combine with molecular probes containing imaging biomarkers. Based on the location and quantification of the probes, information on the reporter genes can be provided indirectly to achieve a much higher diagnostic accuracy. Generally, the reporter genes are active and inducible only in living cells. They are expressed after a specific molecular event such as the addition of the activating substrate [6,8].

**Figure 1:**
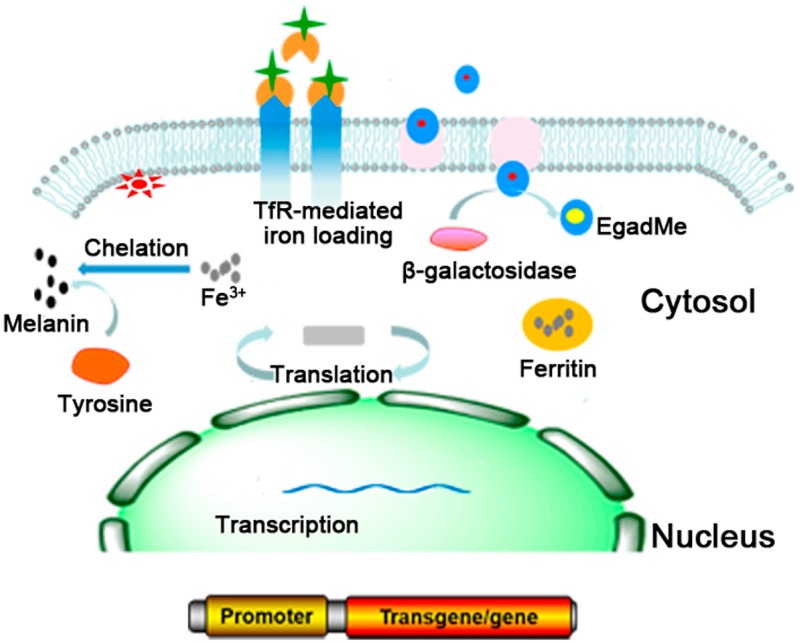
Commonly used MRI reporter genes. Ferritin can specifically bind with iron to provide T_2_ contrast for MRI; The highly expressed TfR in the cells would lead to increased iron uptake to decrease T_2_ relaxation time; LacZ reporter gene expresses β-galactosidase in cells, which increases MR contrast on T_1_ weighted images; TYR reporter encodes tyrosinase to synthesize melanin and provide T_1_ contrast for MRI [21].

MRI reporter genes can be categorized into three classes based on the types of encoded genes: (1) reporter genes encoding an enzyme (e.g., tyrosinase and β-galactosidase); (2) reporter genes encoding the receptor on the cells (e.g., transferrin receptor (TfR)); and (3) endogenous reporter genes (e.g., ferritin reporter gene). The most commonly used MRI reporter genes include ferritin, TfR, tyrosinase (TYR) and β-galactosidase (Table 1). Ferritin can specifically bind with iron, and thus can be used as MR contrast agent on T_2_ weighted images (T_2_WI) [9,10,11]; TfR is highly expressed on the membrane of the target cells that were transferred with TfR reporter gene. Such highly expressed TfR would bring an increased iron uptake in the cells which could result in decreased T_2_ relaxation time [4,12,13]. LacZ reporter gene expresses β-galactosidase in cells, which cleaves off the galactose group of the substrate and exposes a free coordination site of the gadolinium ion. This reaction leads to increased water proton access to the metal ion, resulting in enhanced inner sphere relaxation and increased MR contrast on T_1_ weighted images (T_1_WI) [14]. The TYR reporter gene can be introduced into cells through gene transfer and then expresses tyrosinase in cells which could catalyze the oxidation of tyrosine and dopa and synthesize melanin, which can chelate metal ions (Fe^3+^) to provide T_1_ contrast for MRI [15] (Figure 1).

## 2. Commonly Used MRI Reporter Genes

### 2.1. Ferritin

Ferritin is a ubiquitous intracellular protein that stores iron by specifically binding with iron. Ferritin plays an important role in iron metabolism existing in species ranging from microbes to human beings. In humans, ferritin is mainly distributed in the nucleus of the liver, spleen and brain. The three-dimensional structures of ferritin deduced from different organisms (including human H-chain, horse spleen, rat liver and bacterioferritin of *E.* *coli*, *etc.*) indicate that the ferritin-like proteins have high homology [22,23,24,25,26].

**Figure 2:**
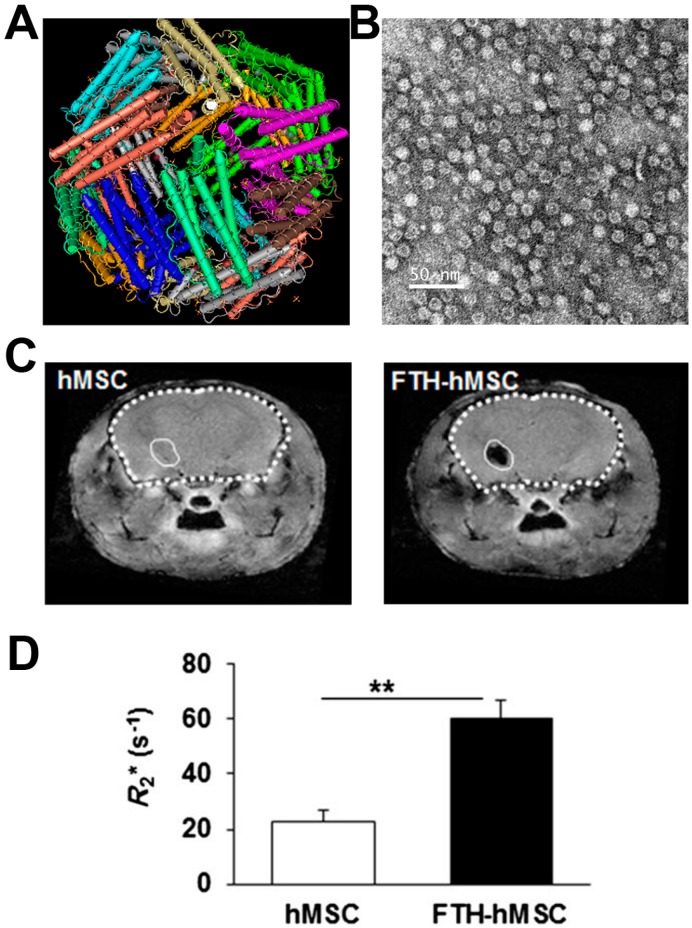
Ferritin reporter gene was used to image human mesenchymal stem cells (hMSCs) by Hoe Suk Kim *et al.* (**A**) The structure of ferritin; (**B**) TEM of ferritin nanocages; (**C**) *In vivo* MRI R_2_* maps of mouse brain transplanted with hMSCs and ferritin reporter gene expressing hMSCs; (**D**) Bar chart showing the average R_2_* values measured from *in vivo* MR images at hMSCs-transplanted sites. Asterisks (**) indicate that the *p* value showed a statistically significant difference (*p* ≤ 0.01). Adapted with permission from [39].

The ferritin gene can be used as a MRI reporter gene to increase the sensitivity of MRI [16]. Ferritin is a heteropolymer assembled from of 24 light and heavy subunits and encapsulated with an iron oxide core (Figure 2A). The ratio of the light and heavy subunits varies among different tissues. The ferritin capsule is a symmetrical spherical void with a diameter around 13 nm (Figure 2B). Both the light and heavy ferritin chain have a similar structure of the 5 A-helix, but they have completely different functions. The heavy chain (FTH1) binds to iron oxide, and it is the major regulator of ferritin activity due to its ferroxidase activity [27,28,29,30,31]. The ferroxidase is responsible for converting labile Fe^2+^ iron to the stabile, insoluble and non-toxic Fe^3+^ form by chelation and neutralization action. By contrast, the light chain has no detectable ferroxidase activity, it just increases the activity of FTH1 and plays an important role in keeping the stability of ferritin. The iron core composed of thousands of ferric oxide ions is located in the center of the protein capsule with a diameter of 7~8 nm.

Iron oxide is a common agent to decrease signal on T_2_-weighted images (T_2_WI) [27]. The R_2_ (R_2_ = 1/T_2_) is directly proportional to iron concentration [22]. The ability of iron-sequester makes ferritin a promising MRI reporter. Its function and storage of labile iron result in high superparamagnetism, which can significantly affect MR relaxation times [32,33]. Thus, cells transfected with a ferritin reporter gene can overexpress ferritin, resulting in the capture of extracellular/endogenous iron to form crystalline iron that is superparamagnetic—this produces detectable contrast on MRI.

Unlike nanoparticle-based techniques (e.g., superparamagnetic iron oxide particles (SPIOs)), ferritin reporter genes would not be diluted with cell division, making them a perfect way to track target cells by MRI. The stable MRI signal is contributed to the continuous production of FTH1 in the daughter cells [34,35,36]. The iron is stored in ferritin as the ferric (Fe^3+^) form, which does not take part in the Fenton reaction, which makes it non-toxic to the organism. Ferritin also can protect the host cells from oxygen and its radical products [32]. Ziv K *et al.* followed chronically overexpressed FTH1 in hepatocytes for 2 years to investigate the long-term effect of over-expressed ferritin on the mice and their MR signal. They found that mice with an elevated level of h-ferritin have increased R_2_ values on MRI *vs.* the control group. Meanwhile, there was no obvious toxicity in livers or other organs in ferritin overexpressing mice. These studies suggested that the ferritin reporter gene is safe and suitable for MR imaging [37]. Unlike other MRI reporter genes such as TfR, TYR, or β-galactosidase, ferritin does not require ectogenic probes. This provides more opportunity to improve the contrast on MRI compared to injected probes which have to overcome the biological barriers. Meanwhile, for injected probes, the clearance of the probes from the blood and nonspecific tissues also causes great obstacles.

Ferritin can also be used to monitor tumor growth and improve diagnostic efficiency and accelerate the development of gene therapies [38]. In addition, there are other applications of the ferritin reporter gene such as non-invasive visualization of neuroblast migration, monitoring event-related promoter activity, and tracking cellular therapeutics, *etc.* [6,39]. For example, Baekelandt *et al.* reported that ferritin overexpression in the rodent brain resulted in significantly enhanced contrast-to-background on T_2_*-weighted MRI [32]. Recently, a new chimeric ferritin molecule engineered with fusing the L and H subunit showed excellent MRI contrast enhancement because of a higher iron loading than the wild-type ferritin [6]. Furthermore, ferritin has been widely used to image neuroblast migration and it can be developed as a switchable approach to imaging glioma cells [40]. Hoe Suk Kim *et al.* transplanted human ferritin heavy chain human mesenchymal stem cells (hMSCs) in the mouse brain. The transduction of FTH led to a significant increase in R2* values [39] (Figure 2C,D). However, the effects of intracellular chelation of iron are decreased with ferritin concentration decrease during cell division. The reduced iron concentration usually results in a poor MR imaging ability as the MRI image quality depends on iron loading index [23,27].

### 2.2. TfR

The TfR reporter gene is another commonly-used reporter gene in MRI imaging. It encodes the cell-surface transferrin receptor once transfected into the target cells. TfR is a homodimer transmembrane glycoprotein with an overall molecular weight of 170–200 kDa. The transferrin receptors widely exist in almost all mammalian cells and they can bind with the transferrin protein [12,41]. The iron in blood exists in the form of transferrin. When the TfR binds with two iron-loaded transferrin molecules to form the TfR-Tf-Fe complex, it will be rapidly internalized through endocytosis. In the intracellular acidic endosomes, iron releases from the TfR-Tf-Fe complex [42,43,44]. Generally, the TfR is regulated by two major factors including cellular iron status and cell growth.

The high expression of TfR increases iron uptake, which will decrease T_2_ relaxation time [45,46]. Recently, Patrick PS *et al.* demonstrated the potential of Timd2 gene for MR imaging with large increases in R2 [47]. In another study, Pereira *et al.* also evaluated the effectiveness of TfR-1 as an MRI reporter gene in mesenchymal stem cells (MSCs), and it was found little differences between cells overexpressing TfR1 transgene and cells incubated with ferric citrate in the short term. However, TfR1 gene-expressing MSCs, but not control cells, showed excellent contrast on MRI in the long term [48,49]. Furthermore, TfR can also be modified to increased T_1_ contrast for MR imaging. For example, Korkusuz *et al.* used novel transferrin-coated gadolinium nanoparticles as MRI contrast agents for brain imaging [50]. Although the TfR reporter gene provides MRI contrast, there is concern that overexpression of the TfR gene and iron accumulation could induce cell toxicity [51]. Thus, the application of TfR needs further evaluation.

### 2.3. TYR

TYR is an intracellular enzyme with catecholase activity. It plays a key role in the melanin production pathway and can also be used as a MRI reporter gene. TYR is the primary enzyme responsible for tyrosine-based melanin production. It catalyzes two fundamental reactions during melanin synthesis. First, tyrosinase produces dopamine by catalyzing the hydroxylation reaction of tyrosine. It can also spontaneously cyclize, oxidoreduce, and polymerize to produce melanin. Generally, tyrsinase alone is sufficient in non-melanogenic cells to catalyze the melanin production [52]. However, the enhanced enzyme activity in cells transfected with the tyroinase gene could lead to a significant increase in signal intensity for monitoring the transfected cells by MRI [18,21].

Melanin widely exists in human hair, skin and other organs. It has a high iron-chelating capacity to paramagnetic ions such as Fe^3+^, Mg^2+^ and Ca^2+^. This results in enhanced MR signals of cells expressing the tryosinase reporter gene [53,54]. The high iron-chelating capacity realized is due to the channels within the melanin granules. This gives melanin the ability to transport metal ions [52,55]. Interestingly, there is no significant evidence of breakdown of melanin, meaning that melanin is a powerful paramagnetic cationic chelator. Based on those unique characters, Weissleder [18] and Enochs [55] *et al.* developed TYR as a MR imaging reporter gene. They demonstrated that the enough concentration of metallomelanin is the reason for the reduced T_1_ relaxivity [53]. Therefore, TYR is widely used as a MRI reporter gene as it has good binding capability of melanin with ions. The ions bound to melanin could create a substantial increase in T_1_ signals. This produces good MRI contrast. Qin *et al.* also successfully demonstrated that the tyrosinase can be applied as a MRI reporter gene both *in vitro* and *in vivo* [52].

Producing too much melanin would certainly be harmful to cell function, and the tyrosinase reporter gene must be regulated by specific promoters. Alfke *et al.* designed and evaluated a contrast agent that could be effectively regulated by the expression of tyrosinase reporter gene under control of the tetracycline response element. This made it possible to identify the tyrosinase gene expression in genetically identical cell clones [53]. Tyrosinase’s sensitivity is dependent on several factors including the total number and the tyrosinase (melanin) expression level of target cells, the depth of tyrosinase expressing tissue, tissue composition between the TYR-expressing cells and the detector, *etc.* [54].

Using TYR as a reporter gene offers many advantages. First, the TYR reporter gene can also be used as a powerful tool in photoacoustic imaging and PET imaging. For example, Qin *et al.* evaluated the TYR gene for PAI, MRI and PET imaging [52]. With photoacoustic imaging, there is no need to add additional contrast agents. However, the photoacoustic microscopy technology suffers from limited imaging depth. MRI is much more appropriate for imaging deeper tissues using TYE reporter gene [54]. TYR expression is inducible, which is very useful in minimizing TYR toxicity. This feature also makes it superior to the expression of constituent reporter genes. In order to reduce the risk of nonspecific side effects brought by overexpression of TYR, the expression of TYR must be limited. This feature will be crucial for future TYR *in vivo* imaging. TYR also can cause several defects even its expression was under control. The most important one is that melanin, the product of TYR, would exist in the cells for a considerable long time even when the TYR reporter gene is turned off [53,54].

In addition to the long persistence time of melanin, the TYR has other pitfalls as well. One is that melanin and melanin precursors can catalyze and bind iron resulting in the production of highly reactive oxygen species that can cause toxic effects [21]. TYR expression can be visualized by MRI implying that TYR can be used a MRI reporter gene. Technically, MRI reporter genes should have an especially low molecular weight, but the TYR reporter gene is currently larger than expected. Thirdly, the TYR currently used has a relatively low expression, which limits its utility in MR imaging.

### 2.4. β-Galactosidase

The β-galactosidase is an indirect imaging reporter gene which is encoded by bacterial LacZ gene. The β-galactosidase catalyzes the substrate X-gal into a Prussian Blue-like product making it detectable with light microscopy [21,53]. β-galactosidase has many advantages, and it is one of the most widely used reporter genes for detection of cell survival and proliferation. For example, β-galactosidase has a large panel of chromogenic and fluorogenic substrates making it a proper candidate for optical imaging [56,57]. However, β-galactosidase is barely expressed in most mammalian tissues and cells [19].

The MR imaging of β-galactosidase usually requires extra contrast agents. One of the most commonly used contrast agent is EgadMe (short for (1-(2-(b-galactopyranosyloxy)propyl)-4,7,10-tris(carboxymethyl)-1,4,7,10-tetraazacyclododecane)gadolinium(III)). EgadMe is a paramagnetic Gd^3+^ chelate. The EgadMe chelator has a high binding affinity for gadolinium, which occupies eight of the nine coordination sites on gadolinium. Normally, the EgadMe is ‘inactive’ and it barely has any influence on T_1_ signal without β-galactosidase, because the water channel is blocked by galactopyranose. However, once the β-galactosidase is expressed *in vivo*, it can catalyze EgadMe to a free coordination site on the gadolinium ion. This causes an irreversible transition of the probe from a water-inaccessible formulation to an ‘active’ state. When the gadolinium ion is transitioned to a water-accessible state, it will generate a strong T_1_ contrast on MR images [19].

There are also some limitations in using EgadMe as an MRI contrast agent. For example, the cleavage rate of EgadMe by β-galactosidase is relative low and EgadMe can be rapidly cleared through renal excretion after injection. Furthermore, the probes would be diluted with cell division leading to a reduction in MR contrast. To overcome this problem, Arena *et al.* designed an MRI probe named Gd-DOTAtyr-gal, which can be used to specifically monitor the β-galactosidase expression in melanoma cells. This novel contrast agent can undergo aggregation and further enhanced T_1_ relaxation following reaction with native tyrosinase resulting in a selective higher signal enhancement [58].

**Figure 3:**
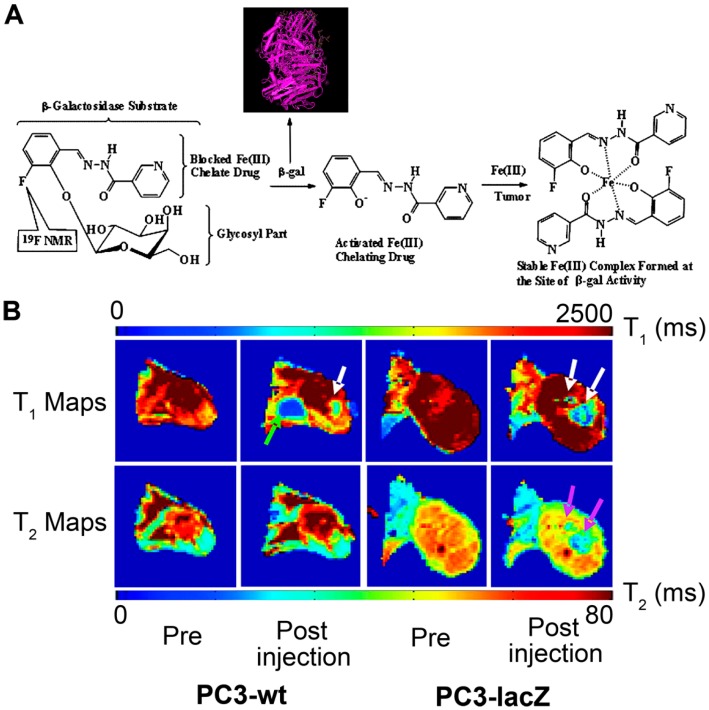
A novel dual ^1^H/^19^F MRI reporter molecule was developed by Yu J *et al.* for *in vivo* detection of β-galactosidase. (**A**) Dual ^1^H/^19^F nuclear magnetic resonance (NMR) gene reporter molecule and (**B**) *In vivo* detection of β-galactosidase [60].

Taking advantage of the specificity of the enzymatic activity in the tissues, Chang *et al.* designed and synthesized a β-galactopyranose-containing gadolinium(III) complex [Gd(DOTA-FPG)(H_2_O)] as a smart contrast agent for MRI. It was found that the T_1_ change percentage of [Gd(DOTA-FPG)(H_2_O)] decreased dramatically with β-galactosidase, and the fold change is much higher than that of Egad- [Gd(DOTA-FPG)(H_2_O)]. This provides a higher-intensity enhancement on MRI images of β-galactosidase gene expression cells than that of non-expression cells [59]. The [Gd(DOTA-FPG)(H_2_O)] is a promising candidate contrast agent for lacZ reporter gene MR imaging [58]. Recently, Yu *et al.* reported a novel dual ^1^H/^19^F MRI reporter molecule for *in vivo* detection of β-galactosidase. The ^19^F nuclear magnetic resonance (NMR) signal was sensitive to β-galactosidase and the liberated aglycone. As a result, the novel molecule can detect both the substrate and the product, and enhance the confidence of enzyme detection (Figure 3) [60].

### 2.5. Lysine Rich-Protein (LRP)

It has been reported that poly-l-lysine has a uniquely high chemical exchange rate, and as a new class of contrast agent, it can be exploited in the chemical exchange saturation transfer (CEST) mechanism to enable detection of dilute solutes [61,62,63,64]. CEST agents are new MRI contrast agents to image a variety of physiological parameters such as pH [65], metabolites, and various diseases (tumor [66,67], and ischemic stroke [68]). The mechanism of CEST is that the exchangeable protons have a chemical shift distinct from water and these protons are selectively saturated. The saturation is transferred to the bulk water *via* chemical exchange. Briefly, following selective radio frequency (RF) irradiation, mobile solute protons are saturated and exchange with the surrounding water molecules to decrease bulk water signal. This RF irradiation allows CEST imaging to be switchable and with the ability of changing the image contrast “on” and “off” via an RF pre-saturation pulse agents.

**Figure 4:**
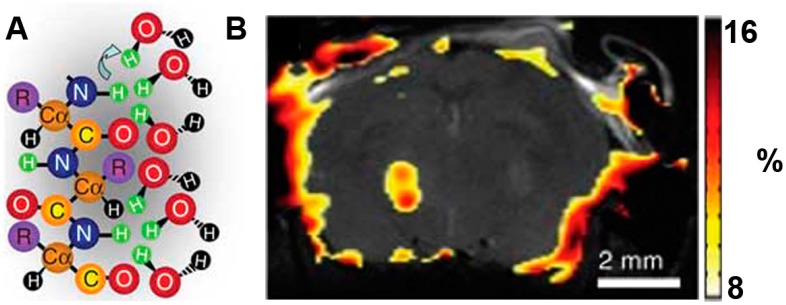
LRP reporter gene imaging of glioma based on CEST MRI. (**A**) Frequency-selective radiofrequency pulses label the amide protons (green); (**B**) Signal intensity-difference map of phantoms. LRP-transfected rat glioma cells were confirmed by Gilad A *et al.* in the overlaid CEST SI difference map [20].

In recent years, researchers have developed many CEST MRI reporter genes [20,69]. For example, a novel artificial gene, lysine-rich protein (LRP) with a high percent of lysine residues was designed to image the transgene overexpression of the glioma cells *in vivo*. Frequency-selective radiofrequency pulses then label the amide protons and reduce exchange with water protons. This decreases MRI signal intensity [20] (Figure 4). Although CEST is a new detection method and offers ways to image embedded exchange and relaxation mechanisms in tissue, the energy deposition in tissue (specific absorption rate: SAR) and the millimolar sensitivity of the technique still limit its clinical use. Generally, a reporter gene encodes to a longer protein. This will give a higher CEST signal via a commercially available full-length synthetic gene. The reporter targets with reduced charge and increases the exchange rate to allow increased detection sensitivity. In addition, frequency-specific reporter genes may be designed to include imino, amine and guanidine protons. This suggests that multiple labeling is possible. It is reasonable to expect that CEST will soon become a standard clinical protocol [20].

## 3. Multimodality Imaging Reporter Genes

The common imaging modalities include MRI, optical imaging (OI) and nuclear imaging. Each has unique strengths and weaknesses (Table 2). MRI offers deep tissue imaging with high spatial resolution and it can simultaneously record anatomic and physiological information [70]. However, MR imaging has low sensitivity and limited utility in molecular imaging. By contrast, optical imaging is a more sensitive tool for molecular imaging. Optical imaging uses nonionizing radiation in the visible and near-infrared wavelengths (400–1500 nm). Its disadvantages include the relatively low spatial resolution and limited depth penetration [71]. Nuclear imaging also has high sensitivity, but it suffers from several disadvantages such as radiation damage, limited exposure time, and relatively poor spatial resolution [72]. The combination of several molecular imaging strategies can harness the strengths of each modality and offer synergistic preponderance over any modality alone with complementary information about molecular and cellular processes [4,73,74,75,76].

An ideal reporter gene for multimodality imaging should offer fast, quantitative, reversible, and intense gene expression-dependent signal contrast [77]. Multimodality reporter genes can be constructed by confusing two or more reporter genes. For example, reporter gene constructed by confusing luciferase and thymidine kinase allows imaging with bioluminescence and PET, respectively. The fusion reporter genes are joined together with short linkers and controlled with a single promoter [78,79]. Fusion reporter gene imaging offers the complementary advantages of both modalities and eliminates the disadvantages of single imaging modalities. Furthermore, multimodality imaging provides a more flexible method and allows selection of the appropriate imaging technologies to solve the biomedical problem (Table 3).

### 3.1. Single Reporter Genes for Multimodality Imaging

Some single reporter gene can be imaged using several modalities simultaneously. This kind of reporter gene has many advantages over fusion reporter genes. In the first place, there is no need to worry about the change in reporter gene properties due to the reporter gene confusion, because activity attenuation may occur in fusion multimodality genes. Secondly, the construction of a single reporter gene is much more convenient than fusion reporter genes [56,84].

#### 3.1.1. The Organic Anion Transporting Polypeptides (Oatp) 1

**Figure 5:**
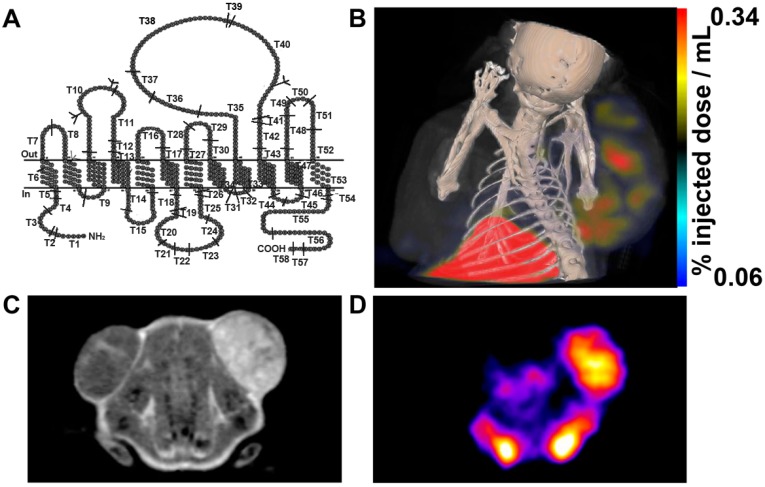
Oatp1 expression was detected using MRI and SPECT by Drs. Patrick and Xiao *et al.* (**A**) Predicted structure of oatp1a1; (**B**) SPECT-CT image of ^111^In-EOB-DTPA injected mouse. The xenograft on the left flank was a control and the xenograft on the right flank expressed Oatp1; (**C**) T_1_-weighted MR image of Gd-EOB-DTPA injected mouse; (**D**) SPECT image of the ^111^In-EOB-DTPA injected mouse [79,85].

Oatp1 is a member of the SLCO superfamily and it transports a variety of imageable small molecules across the plasma membrane (Figure 5A). Oatp1 is known as Slc21a1 or Slco1a1. The oatp1 is mainly expressed in the kidney and liver. It can serve as a reporter gene and produce a rapid, intense, reversible signal enhancement in T_1_-weighted MR imaging by transferring MRI contrast agent (e.g., gadolinium ethoxybenzyl-DTPA (Gd-EOB-DTPA)) [79,80]. The oatp1-based reporters have been used for tracking implanted stem cells and monitoring expression of gene therapy vectors with maximum signal enhancement. Interestingly, the Gd^3+^-based contrast agent can be imaged with both SPECT and MRI by exchanging Gd^3+^ for ^111^In [79] (Figure 5B–D).

The Oatp1 gene has several advantages in molecular imaging. Firstly, Oatp1 can use Gd^3+^ as a MRI contrast agent, which is a positive contrast agent on MRI and offers an easily detected signal in T_1_-weighted images. Secondly, the contrast agent is small. Thirdly, the Oatp1 shows restricted tissue expression, and the background uptake in most tissues is expected to be low. The observed relationship between R_1_ and induction of Oatp1 expression indicates that the kinetics of contrast agent uptake could give quantitative estimates of gene expression levels. Fourthly, unlike SPIO-based reporter genes, the signal intensity of Oatp1 reporter genes will not be diluted with cell division or exocytosis. The Gd^3+^-based contrast agents have been widely used in clinical imaging, and Oatp1 gene imaging strategy would be easier to translate into clinical applications.

#### 3.1.2. LacZ

LacZ is a commonly used reporter gene for MR imaging. High quality MRI images can be obtained by using proper MRI contrast agents like EgadMe. Besides EgadMe for lacZ reporter gene MR imaging, other contrast agents can also be used for different imaging modalities. The β-galactosidase can serve as an activating contrast agent to visualize the changes in lacZ gene expression over time [4]. Recently, near-infrared fluorescent β-galactosidase-responsive probes have been developed to visualize lacZ gene expression. This can be activated upon cleavage by β-galactosidase [5]. For example, DDAOG, a β-galactosidase substrate whose cleavage product has detectable far-red fluorescence properties, is a promising contrast agent in deep tissue imaging with optics. Importantly, DDAOG and its cleavage product can be detected simultaneously, and the prominent fluorescence enhancement can be observed in 9L-lacZ tumor region via DDAOG contrast agents [56].

#### 3.1.3. TYR

TYR is not only a MRI reporter gene, but also can be detected by photoacoustic imaging (PAI) and PET imaging [52]. PAI is also called optoacoustic or thermoacoustic imaging. It is based on the photoacoustic (PA) effect. The target tissues can generate acoustic waves after the absorption of electromagnetic energy. Optical and radio-frequency waves can promote target tissues to generate acoustic waves [81]. When the target tissues have absorbed the incident light and converted it to heat, it will result in local thermoelastic expansion. The expansion will be further propagated as an ultrasonic wave [82]. *Vs.* other optical imaging techniques, photoacoustic imaging offers higher spatial resolution and higher contrast. This allows deep tissue imaging [86]. Photoacoustic tomography is sensitive to melanin which is product of the TYR gene. Melanin is commonly existed in the skin, and it is one of the primary absorbers. Melanin also has a broad absorption spectrum in the visible range and strong tissue penetrating properties. This provides strong absorption contrast for PAI. When introducing the TYR gene into non-melanin expressing tissues, melanin production will be induced. Thus, the TYR gene can be used as a reporter gene to yield melanin contrast for PAI [82].

**Figure 6:**
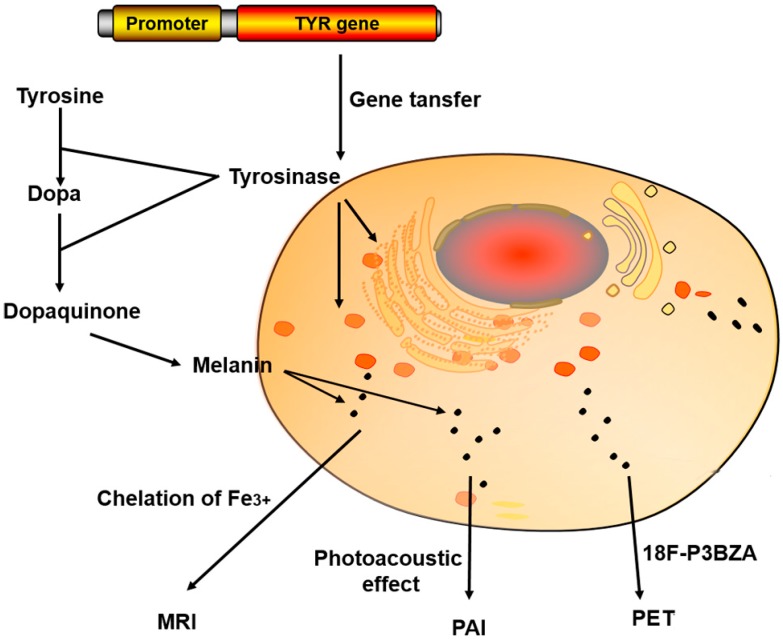
TYR reporter gene is transfected into cells, and the expressed tyrosinase catalyzes the oxidation of tyrosine and Dopa to melanin, which can serve as a multi-functional target for photoacoustic, MRI, and PET imaging [52].

Generally, melanin can strongly bind with many small molecules containing aromatic structures both *in vitro* and *in vivo* to produce signals on PET images. For example, the introduction of melanin-avid probes such as ^18^F-Fluoro-2-deoxy-d-glucose (^18^F-FDG) and ^123^I-*N*-(2-diethylaminoethyl 4-iodobenzamide) benzamide (^123^I-BZA) can perform TYR-based PET imaging. Therefore, The TYR gene can be effectively used as a PET reporter gene by detecting melanin [83]. Those PET reporter probes have many favorable features like high radiochemical yield, high tumor uptake, and simple labeling procedures. Recently, Qin *et al.* evaluated a TYR reporter gene as a stand-alone imaging reporter gene for PAI/MRI/PET imaging. They transfected MCF-7 cells with plasmids encoding TYR and imaged the tyrosinase expressing MCF-7 cells using melanin-targeted *N*-(2-(diethylamino)ethyl)-^18^F-5-fluoropicolinamide as a PET reporter probe. The results showed that the TYR-expressing MCF-7 cells had much better signal on PAI/MRI/PET images [52,83] (Figure 6).

There are many advantages to using TYR single reporter gene to provide multimodality images. First, the structure of TYR is very simple. Second, the expression rate of TYR is at a high level. Third, the TYR expression is controllable. Last but not the least, there are little side effects when using TYR reporter gene, because melanin is already a very common substance that widely exists in human cells and tissues [52].

#### 3.1.4. Combination of Single Reporter Gene and Contrast Agents for Multimodality Imaging

This strategy combined a single reporter gene and one contrast agent for multimodality imaging. This is much convenient than fusion reporter genes, because the problems of fusion protein expression and activity change are not a concern any more. Furthermore, it allows visualization of cellular and subcellular processes in individual cell and tissue by two modalities. However, target cells cannot be tracked longitudinally by this strategy *vs.* single gene and fusion gene multimodality imaging systems, because the concentration of the contrast agents will be diluted rapidly with cell death and phagocytosis [4,74,87].

The imaging systems combining reporter gene with contrast agents for multimodality imaging have been widely used for monitoring cell engraftment and survival. For example, in cardiac cell transplantation therapy, Higuchi *et al.* retrovirally transduced the sodium iodide symporter (NIS) gene into human endothelial progenitor cells (HEPCs) that is derived from CD34^+^ mononuclear cells of umbilical cord blood. The labeled HEPCs can be visualized by both MRI and PET. The HEPCs remain active after transduction, and the reporter protein is stably expressed in target cells, which indicated that it was a viable way to track target cells [84]. Because of the dilution of MRI contrast agents, MRI can only offer cell localization information soon after transplantation. However, the signal intensity of NIS reporter gene did not decrease with time. This confirms that it is a good complement to MRI. Thus, effective visualization of survival of transplanted cells by this bimodality imaging system would efficiently evaluate the cell transplantation and therapeutic effects.

### 3.2. Fusion Reporter Gene Used for Multimodality Imaging

Many reporter genes can be imaged by several different imaging modalities with advantages over fusion reporter genes such as relatively stable properties, convenient acquisition, and easier construction. But single reporter genes for multimodality molecular imaging just accounts only a small percentage of the multimodality imaging field. They still suffer from various limitations including low sensitivity, spatial resolution and limited depth penetration. *Vs.* single reporter genes for multimodality imaging, fusion multimodality reporter genes can be constructed by confusing two or several reporter to enable highly sensitive detection and overcome many shortcomings of each modality alone [88,89]. This plays an important role in monitoring disease progression and therapy [90]. However, the fusion reporter gene should be consistent with each individual reporter protein. Meanwhile, the fusion protein should be stable and not disintegrate [90]. In addition, the signal intensity of the fusion reporter genes may be attenuated from one or more of the components *vs.* single reporter genes [91].

Both fluorescent and bioluminescent proteins have been widely used as reporter genes for molecular imaging. Optical imaging is a sensitive imaging method, but its application is limited in peripheral organs like skin because of low light penetration. Lots of MRI reporter genes have been proved to be useful in clinical research. The combination of optical reporter gene and MRI reporter gene can provide noninvasive images with high sensitivity, spatial resolution and deep penetration depth for biological processes. The MRI and optical imaging of target cells/tissues at molecular/cellular levels can be performed simultaneously using the bimodality reporter gene imaging system. This fusion reporter gene offers a more effective way to image the exact localization, extent, and metabolic activity of the target molecular in deep tissues [74].

Kim *et al.* constructed a fusion reporter gene by combining a human ferritin [myc-tagged human ferritin heavy chain (myc-hFTH)] gene and green fluorescent protein (GFP) gene. The transgene construct was stably transfected into MCF-7 cells which were transplanted into mice or rats. The results showed that myc-hFTH tumors had significantly higher fluorescence signal and lower signal intensities in T_2_-weighted MRI than mock-transfected controls [74]. Taking advantage of ferritin reporter genes, there is no need to inject MRI contrast agents to subjects. Unpleasant side effects including toxicity, allergic reactions, *etc.* can be significantly reduced. Furthermore, long-term imaging can be performed with ferritin MRI reporter gene. Similarly, Choi *et al.* noninvasively imaged and quantified the metastatic melanoma cells in the lymph nodes with the same fusion reporter gene in B16F10 murine melanoma models [38]. Recently, Ono *et al.* also successfully developed dual-reporter ferritin-DsRed under a β-actin promoter and monitored gene expression by MRI and optical imaging in a brain tumor model [27].

## 4. Conclusions and Perspectives

Molecular imaging provides a novel noninvasive method to real-time monitor cellular function and molecular processes in living organisms. In both preclinical and clinical studies, MRI excels at imaging tissue deep, high resolution and excellent soft tissue contrast, but suffers from modest sensitivity. To achieve molecular imaging of disease biomarkers, MRI probes with high specificity and high sensitivity are required. Reporter gene based on MRI has led to important discoveries regarding cell migration, retention and survival. At the same time, structural and functional MRI can be used for therapy monitoring of long-term gene expression from living organisms. Currently the field of molecular MRI is undergoing rapid development. Lentiviral vector mediated therapeutic and MRI reporter gene delivery to target organs/tissues/cells has great potential for the future treatment. Importantly, MRI is a widespread clinical imaging modality, and structural MRI scans are already part of cancer diagnosis and progression monitoring. The development of MRI reporter genes would play an important role in translational medicine.

Each molecular imaging modality has its intrinsic advantages and limitations. The utilization of multimodality molecular imaging has promising prospects for basic research and clinical applications. Thus, coupling MRI reporters with bioluminescent, fluorescent, and/or PET reporters will be commonplace. It is very important to establish proper evaluation methods with the aid of multimodality molecular imaging in preclinical studies and bench to bedside translation. Furthermore, the development of new MRI reporters can increase the signal intensity for more reliable imaging analysis. Combined with multimodality imaging capabilities, the reporter would provide more detailed and accurate pathological information about the biological processes at molecular levels. On the other side, the technical advances in MRI devices would be helpful for the translation of advanced MRI reporter gene into the clinic.

## Figures and Tables

**Table 1:** Commonly used MRI reporter genes.

Gene/Protein	Contrast Mechanism	Observed Change	Test System	Ref.
Ferritin	Sequesters iron from labile intracellular iron pool and acts as an intracellular SPIO analog	T_2_ changes from 45 to 20 ms with 30 µg Ferritin expressed per mg of total protein (14 T)	Cell culture; viral-mediated transfection in mouse brain	[16]
Transferrin receptor (TfR)	Transferrin-conjugated SPIO particles are internalized by ectopically expressed TfR on transfected cells	50% change in T_2_-weighted MRI signal (3 mg iron injected per mouse, 7.1 T)	Mice implanted with TfR-expressing gliosarcoma cells	[17]
Tyrosinase (TYR)	TYR produces melanin to chelate metal ions (Fe^3+^)	37% increase in T_1_-weighted MRI signal (1.5 T)	Transfected mouse fibroblasts and HEK cells	[18]
β-galactosidase	Expressed β-gal cleaves a caged synthetic Gd^3+^ compounds	60% T_1_-weighted signal increase (3.2 nmol per frog embryo, 12 T)	Xenopuslaevisembryos transfected withLacZ	[19]
Lysine rich-protein (LRP)	Chemical exchange saturation transfer (CEST)	134% signal increase (6 days after 5 × 10^4^ cells transplanted into the striatum of NOD-SCID male mice, 11.7 T)	Mice implanted with LRP and EGFP expressing xenografts respectively in opposite hemispheres	[20]

**Table 2:** Commonly used *in vivo* small-animal imaging modalities.

Molecular Imaging Methods	Resolution	Depth	Sensitivity	Cost	Potential Clinical Uses
OI	1–5 mm	up to < 5 cm	10^−9^–10^−12^ mol/L	low cost	very low
MRI	10–100 µm	no limit	10^−3^–10^−5^ mol/L	high cost	yes
PET	1–2 mm	no limit	10^−10^–10^−12^ mol/L	high cost	yes
SPECT	0.3–1 mm	no limit	10^−10^–10^−12^ mol/L	high cost	yes

**Table 3:** Reporter genes used for multimodality imaging.

Single/Fusion Reporter Gene	Gene/Protein	Contrast Mechanism	Imaging Method	Ref.
Single reporter gene	Oatp1	(1) Transfer MRI contrast agents (2) Mediate uptake of Gd^3+^ or ^111^In based hepatotrophic contrast agents	MRI (T_1_WI) SPECT	[79,80]
	LacZ	(1) Express β-gal to cleave Gd^3+^ compounds (2) Visualize lacZ gene expression with activated fluorescent contrast agents	MRI (T_1_WI) NIR	[4,5,56]
	TYR	(1) Broad optical absorption for photoacoustic effect (2) Chelate metal ions (Fe^3+^) providing contrast for MRI (3) Melanin-avid PET probes, ^18^F-P3BZA	PAI MRI (T_2_WI) PET	[52,81,82,83]
Multimodality reporter gene	Single reporter gene and contrast agent	Sodium iodide symporter (NIS) gene and iron oxide	PET MRI	[84]
	Fusion reporter gene	Ferritin gene fused with green fluorescent protein gene	MRI NIR	[74]
